# The Effects of Inhalation Aromatherapy on Anxiety in Patients With Myocardial Infarction: A Randomized Clinical Trial

**DOI:** 10.5812/ircmj.15485

**Published:** 2014-08-05

**Authors:** Zahra Najafi, Mohsen Taghadosi, Khadijeh Sharifi, Alireza Farrokhian, Zahra Tagharrobi

**Affiliations:** 1 Department of Health Management, Faculty of Nursing and Midwifery, Kashan University of Medical Sciences, Kashan, IR Iran; 2 Department of Medical Surgical Nursing, Faculty of Nursing, Kashan University of Medical Sciences, Kashan, IR Iran; 3 Department of Internal Medicine, Faculty of Medicine, Kashan University of Medical Sciences, Kashan, IR Iran

**Keywords:** Anxiety, Aromatherapy, Lavender, Myocardial Infarction

## Abstract

**Background:**

Anxiety is an important mental health problem in patients with cardiac disease. Anxiety reduces patients’ quality of life and increases the risk of different cardiac complications.

**Objectives:**

The aim of this study was to investigate the effects of inhalation aromatherapy on anxiety in patients with myocardial infarction.

**Patients and Methods:**

This was a randomized clinical trial conduced on 68 patients with myocardial infarction hospitalized in coronary care units of a large-scale teaching hospital affiliated to Kashan University of Medical Sciences, Kashan, Iran in 2013. By using the block randomization technique, patients were randomly assigned to experimental (33 patients receiving inhalation aromatherapy with lavender aroma twice a day for two subsequent days) and control (35 patients receiving routine care of study setting including no aromatherapy) groups. At the beginning of study and twenty minutes after each aromatherapy session, anxiety state of patients was assessed using the Spielberger’s State Anxiety Inventory. Data was analyzed using SPSS v. 16.0. We used Chi-square, Fisher’s exact, independent-samples T-test and repeated measures analysis of variance to analyze the study data.

**Results:**

The study groups did not differ significantly regarding baseline anxiety mean and demographic characteristics. However, after the administration of aromatherapy, anxiety mean in the experimental group was significantly lower than the control group.

**Conclusions:**

Inhalation aromatherapy with lavender aroma can reduce anxiety in patients with myocardial infarction. Consequently, healthcare providers, particularly nurses, can use this strategy to improve postmyocardial infarction anxiety management.

## 1. Background

Myocardial Infarction (MI) is the most serious complication of coronary artery disease (CAD) and one of the most common health problems worldwide. CAD has received considerable attention because of its serious adverse effects (1, 2). Mortality rates of cardiovascular diseases (CVD) in developed and developing countries are 40% and 28%, respectively (3). In our country, Iran, CVD is also the first leading cause of death (4). In 2008, mortality rate of CVD in Iran among men and women were 421 and 348 cases per 100000 people, respectively (5).

Besides a high mortality rate, CVD also causes different personal, familial, social, and economical problems as well as psychological disorders including anxiety, despair, fatigue, decreased self-confidence (6) and depression (7). Anxiety is an important mental health problem in patients with cardiac disease. The prevalence of anxiety in patients with MI is up to 50% (8). Factors such as unfamiliar environment, lack of family support, healthcare costs, fear of death, and uncertainty over the results of diagnostic and medical procedures and disease prognosis are among the most important sources of anxiety in patients with MI (9, 10). Besides, hospitalization in intensive care units creates anxiety for these patients. Bassampoor indicated that most patients experience severe anxiety during the first 48 hours of hospitalization as what found by Kasme and Hallet (11).

Anxiety affects patients’ quality of life negatively and increases the risk of cardiac complications such as ischemia, myocardial infarction, and cardiac arrhythmias (7, 12, 13). It stimulates the sympathetic nervous system (14, 15). Subsequent catecholamine release increases heart rate, blood pressure, strength of cardiac contractions, and myocardial oxygen demand, which in turn raise the risk of life-threatening cardiac dysrhythmias (15). Consequently, anxiety can increase the risk of post-MI death (12, 16). Given its adverse effects on cardiovascular system, anxiety management is a matter of great importance in patients with MI.

Anxiety is managed both pharmacologically and non-pharmacologically (17). Pharmacologic management of anxiety can significantly improve patient outcomes; however, it is associated with different side effects such as fatigue, confusion and restlessness (11). Moreover, despite the presence of different anxiolytic agents, many patients experience anxiety for months (12) or even one year after MI (18). Consequently, non-pharmacologic management of anxiety has recently assumed an added importance.

Aromatherapy is one of the non-pharmacological strategies for anxiety management developed to reduce patients’ anxiety (19). Lavender (*Lavandula angustifolia* and *Lavandula stoechas*, from the Labiatae family) is a plant with an anxiolytic and relaxing aroma. Main components of lavender are linalool a sedative agent which affects gamma-Amino butyric acid (GABA) receptors in the central nervous system (20) and linalyl acetate a narcotic agent (21). Previous studies confirmed the positive effects of lavender on examination anxiety (22), delivery anxiety in primiparous women (23) and hemodialysis-related anxiety (24). However, controversies exist regarding the anxiolytic effects of lavender. Brooker et al. found that aromatherapy had no effect on agitation in patients with dementia (25). Graham et al. also reported that inhalation aromatherapy did not relieve radiotherapy-related anxiety (26).

No study was found regarding the effects of aromatherapy on anxiety of patients with MI in available databases. Moreover, despite serious side effects of anxiolytic agents, dominant anxiety management strategy in Iran is medication therapy. Accordingly, regarding different uses of aromatherapy in nursing care, we conducted this study to assess the effectiveness of inhalation aromatherapy on post-MI anxiety.

## 2. Objectives

The aim of this study was to investigate the effects of inhalation aromatherapy on anxiety in patients with myocardial infarction.

## 3. Patients and Methods

This was a randomized clinical trial conducted in 2013 from February to August. The study population was all patients with MI at their second day of hospitalization. The study setting was all the coronary care units of a general teaching hospital affiliated to Kashan University of Medical Sciences, Kashan, Iran. This public hospital has 536 beds and 30 units which has two CCU units with 14 beds. We calculated the study sample size using the findings of Bassampoor and Hashemzadeh et al. studies (11, 27). Accordingly, with a confidence interval of 95% and a power of 80%, the sample size was determined as 32 patients in each group using the following formula:

$$\frac{2{[z(1-\propto ⁄2)+z(1-\beta \mathrm{)]}}^{2}(\delta^{2})}{d^{2}}$$

To compensate probable attritions, we decided to recruit 35 patients in each group. The inclusion criteria were:

- Age between 20-80 years
- Able to speak Persian
- Established diagnosis of MI
- No previous history of MI
- No indication of cardiopulmonary resuscitation at the time of hospital admission
- No history of addiction, smelling impairments, mental disorder, allergic rhinitis, eczema, respiratory diseases such as asthma, or uncontrolled contagious disease
- Being oriented to person, place, and time
- Having stable vital signs
- Having no pain (any pain) at the time of completing the study questionnaire; and
- Obtaining a score of more than 20 in the Spiel Berger’s State Anxiety Inventory; the minimum score is 20 in SSAI, the score higher than 20 was used to demonstrate the intervention effect.
- Patients who required cardiopulmonary resuscitation or took over the counter tranquilizers and those who developed allergy to lavender aroma or experienced cardiac dysrhythmias, cardiogenic shock, or death during the study were excluded. Researchers recruited patients using the sequential sampling method. Accordingly, researcher explained the purpose and method of study to patients who had inclusion criteria and assured them regarding information confidentiality. Eligible patients signed an informed consent and entered the study. We randomly assigned the study participants to experimental and control groups using the block randomization technique.

Patients in the experimental group received inhalation aromatherapy with lavender aroma twice a day (10-11 AM and 6-7 PM) for two subsequent days (second and third days of hospitalization); these times were selected based on the schedule of treatment and care in related units and the time of patients rest. Each session of aromatherapy lasted for twenty minutes. For each aromatherapy session, researcher poured three drops of lavender essence (prepared from Lavandula stoechas flower buds by Barij Essence Pharmaceutical Company, Kashan, Iran) on a Kleenex and attached it to patient’s collar. Then, researcher asked patient to breathe normally for 20 minutes. Patients in the control group received the routine care of study setting without aromatherapy.

The study instrument consisted of a demographic questionnaire and the Spielberger’s State Anxiety Inventory. The demographic questionnaire included items regarding participants’ age, gender, education, related education to medical sciences, employment, financial and marital status, residence, number of children, insurance coverage, family support, family history of MI, post-MI death among closed relatives, tranquilizer consumption, smoking, known concurrent diseases, and awareness of the reason for hospitalization. We asked 10 nursing lecturers to confirm the content validity of the demographic questionnaire. Moreover, we employed the test-retest technique to evaluate the reliability of the questionnaire. Accordingly, we asked 10 patients to complete the questionnaire twice with a five-day interval. The test-retest correlation coefficient was 1.

To assess patients’ anxiety, we used the 20-item Spiel Berger’s State Anxiety Inventory (SSAI). SSAI demonstrates people’s feelings about the current situation. SSAI is a 4-point Likert scale in which items are scored form 1 (not at all) to 4 (very much). Ten items of SSAI (items 1, 2, 5, 8, 10, 11, 15, 16, 19, and 20) are scored reversely. The total score of inventory ranges from 20 to 80. Higher scores reflect higher levels of state anxiety (28). In Iran, the researchers confirmed the concurrent validity of SSAI. They also evaluated the internal consistency of inventory and reported a Cronbach’s alpha of 0.93 (29). We administered SSAI for patients in the experimental group at the beginning of study (T1) and 20 minutes after each aromatherapy session (T2-T5). Patients in the control group completed the SSAI concurrently with the patients in the experimental group. The questionnaires were filled by using the interview method. 

Data was analyzed using the Statistical Package for Social Sciences (SPSS v. 16.0). We used Chi-square, Fisher’s exact and the independent-samples T-test to compare the study groups for categorical and continuous variables; Kolmogorov Smirnov was used to assess normal distribution of quantitative variables in each group. Moreover, we used the repeated measures analysis of variance (repeated measures ANOVA) test to compare the study groups for the level of anxiety across five measurement time points. The level of significance was set at below 0.05. An Institutional Review Board and Ethics Committee (No: P/29/5/1/3751, Date: 07.01.2013) affiliated to Kashan University of Medical Sciences approved the study. Moreover, the Iranian Registry of Clinical Trials registered the study with the number of IRCT2012112511572N1.

## 4. Results

**Figure 1.**
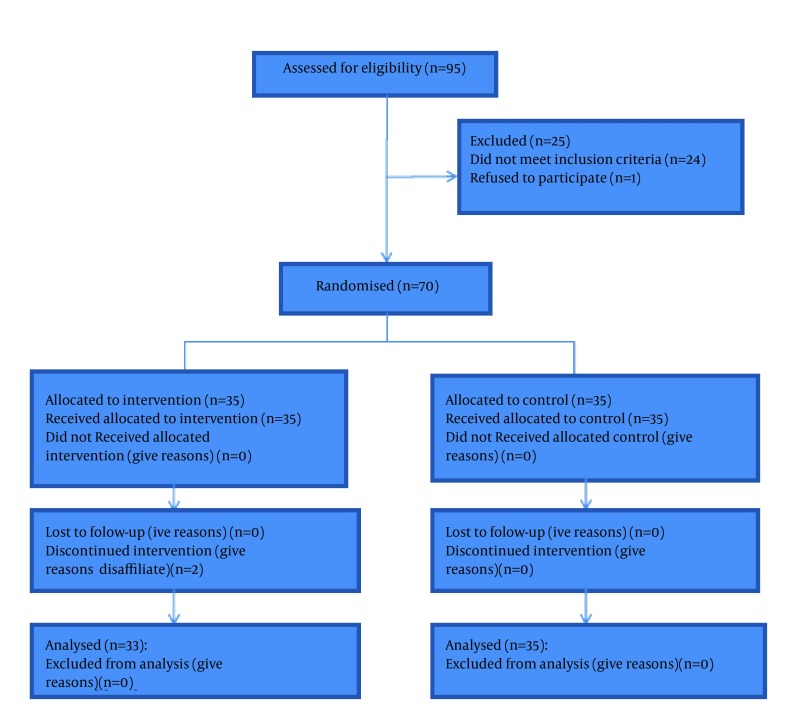
Sampling Process

Totally, 70 patients participated in the study and 35 patients in each group. Two patients in the experimental group were excluded from the study because of lack of cooperation (Figure 1). Chi-square test, Fisher’s exact test, and the independent samples T-test showed that the study groups did not differ significantly regarding demographic and background variables (P < 0.05; Table 1).

Repeated measures ANOVA for the within-subjects factor of time revealed that the levels of anxiety in the experimental group differed significantly between the five measurement time points (Greenhouse-Geisser F = 47.289, P-Value < 0.001). The results of the Bonferroni post-hoc test showed that all differences were statistically significant except for the differences between T1 and T2 and between T3 and T4 (P-Value < 0.05). However, the results of the repeated measures ANOVA for within-subjects factor of time demonstrated that the differences in the levels of anxiety between the five measurement time-points were not statistically significant in the control group (Greenhouse-Geisser F = 2.838, P-Value = 0.060).

**Figure 2.**
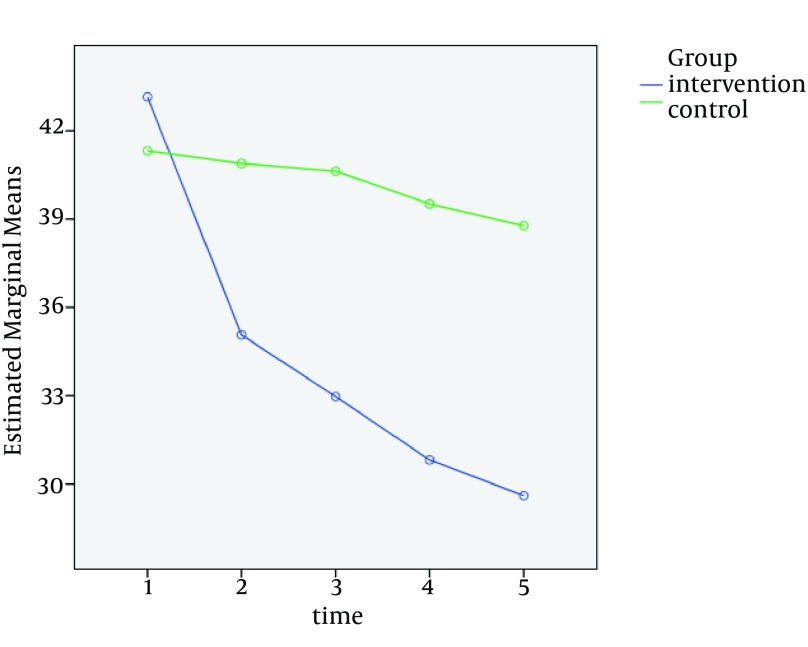
Interactive Effect of Time and Aromatherapy on Anxiety

On the other hand, the results of the repeated measures ANOVA for between-subjects factor of group revealed that the study groups differed significantly between the five measurement time points regarding the level of anxiety (Greenhouse-Geisser F = 21.635, P-Value < 0.001; Figure 2). At the beginning of the study (T1), the mean and the standard deviation of state anxiety were 43.15 ± 12.179 and 41.31 ± 12.890 in the experimental and control groups, respectively. The results of the independent samples T-test revealed that this difference was not statistically significant (P-Value = 0.548; Table 2). However, the results of the same test revealed that the levels of anxiety in the experimental group at T2-T5 were significantly lower than the control group (P-Value < 0.05; Table 2).

**Table 1.** Demographic and Background Status of Participants in Experimental and Control Groups

Variable	Experimental (n =33), No. (%)	Control (n =35), No. (%)	P Value ^a^
**Gender**			0.532 ^b^
Female	9 (27.3)	12 (34.3)	
Male	24 (72.7)	23 (65.7)	
**Education**			0.598 ^b^
Illiterate	12 (36.4)	15 (42.9)	
Under diploma	12 (36.4)	14 (40.0)	
Diploma and Over diploma	9 (27.3)	6 (17.1)	
**Related education to medical sciences**			1.000 ^c^
Yes	1 (50.0)	1 (100)	
No	1 (50.0)	0 (0.0)	
**Employment**			0.154 ^b^
Unemployed	11 (33.3)	14 (40.0)	
Employed	10 (30.3)	4 (11.4)	
Self-employment	12 (36.4)	17 (48.6)	
**Financial status**			0.858 ^b^
Below-average	12 (36.4)	12 (34.3)	
Middle to high	21 (63.6)	23 (65.7)	
**Residence**			0.182 ^b^
Rural	5 (15.2)	10 (28.6)	
Urban	28 (84.8)	25 (71.4)	
**Marital status**			0.663 ^b^
Married	27 (81.8)	30 (85.7)	
Single	6 (18.2)	5 (14.3)	
**Insurance coverage**			0.123 ^b^
Yes	30 (90.9)	27 (77.1)	
No	3 (9.1)	8 (22.9)	
**Family support**			1.000 ^c^
Low	2 (6.1)	3 (8.6)	
Middle to high	31 (93.9)	32 (91.4)	
**Family history of MI**			0.858 ^b^
Yes	12 (36.4)	12 (34.3)	
No	21 (63.6)	33 (65.7)	
**Post-MI death in closed relatives**			0.884 ^b^
Yes	9 (27.3)	9 (25.7)	
No	24 (72.7)	26 (74.3)	
**Tranquilizer consumption**			1.000 ^c^
Yes	32 (97)	34 (97.1)	
No	1 (3.0)	1 (2.9)	
**Smoking**			0.492 ^b^
Yes	12 (36.4)	12 (34.3)	
No	21 (63.6)	33 (65.7)	
**Known concurrent diseases**			0.611 ^b^
Yes	19 (57.6)	18 (51.4)	
No	14 (42.4)	17 (48.6)	
**Awareness of the reason for hospitalization**			0.419 ^b^
Yes	5 (15.2)	8 (22.9)	
No	28 (84.8)	27 (77.1)	
**Age, y**	57.24 ± 13.962	61.54 ± 12.013	0.173 ^d^
**Number of children**	3.24 ± 1.442	3.91 ± 2.837	0.080 ^d^

^a^ Non-Significant.

^b^ Chi-Square.

^c^ Fisher's Exact.

^d^ Independent t-test.

**Table 2.** Comparing the Anxiety Score Between Experimental and Control Groups at First and Second Days

Intervention Time	Experimental (n = 33), Mean ± SD	Control (n = 35), Mean ± SD	t	P Value a
**Pre test**	43.15 ± 12.179	41.31 ± 12.890	0.603	0.548 ^b^
**First day**				
First time	35.06 ± 10.232	40.89 ± 13.217	-2.039	0.046 ^c^
Second time	32.97 ± 8.424	40.63 ± 13.565	-2.814	0.007
**Second day**				
First time	30.82 ± 8.013	39.51 ± 13.969	-3.171	0.002
Second time	29.61 ± 7.318	38.77 ± 13.954	-3.419	0.001

^a^ Independent t-test

^b^ Non-Significant

^c^ Significant

## 5. Discussion

The aim of this study was to investigate the effects of inhalation aromatherapy on anxiety in patients with myocardial infarction. The study findings revealed that inhalation aromatherapy significantly reduced anxiety in patients with MI. This finding is in line with the findings of Itai et al. (30) Lehrner et al. (31) Kutlu et al. (22) and Canaani et al. (24) who reported that inhalation aromatherapy with lavender aroma significantly reduced examination anxiety as well as hemodialysis and dental office related anxiety. However, our findings contradict the findings of Muzzarelli et al. (32) and Graham et al. (26). Muzzarelli et al. reported that the levels of endoscopy-related anxiety did not differ significantly in patients who received lavender aromatherapy compared to those receiving placebo. This contradiction can be related to the shorter five-minute period of aromatherapy in their study. On the other hand, Graham et al. found that the levels of radiotherapy-related anxiety in the placebo group (receiving carrier oil only) were significantly lower than both the experimental groups (receiving either carrier oil with fractionated oils or pure essential oils of lavender bergamot, and cedar). This contradiction may be due to the differences between the design of these two studies (four versus only one session of aromatherapy) and participants’ underlying diseases (MI versus cancer). Oil essences can regulate mood and emotions and reduce anxiety by stimulating olfactory receptors located in the olfactory bulb as well as stimulating the limbic system (33). Lavender exerts its anxiolytic effects presumably through the same mechanism stimulating the limbic system.

Nikfarjam indicated that lavender, like benzodiazepines, exerts its anxiolytic effects through increasing the levels of GABA in amygdala as Cavanagh and Wilkinson. Moreover, the narcotic and sedative effects of linalool and linalyl acetate compounds contained in lavender can relieve anxiety (34). The limitations of the study were inability to transfer patients from critical unit to a suitable environment for performing intervention, unaffected of patients' response at the presence of their family and lack of flexibility blinding. Therefore, it is recommended to perform further studies by considering or if possible removing these limitations.

In conclusion, inhalation aromatherapy with lavender aroma can help relieve post-MI anxiety. Given the paramount importance and high prevalence of anxiety in patients with MI as well as the cost-effectiveness and simplicity of aromatherapy with lavender aroma, healthcare providers, particularly nurses, can use this strategy to improve post-MI anxiety management. Moreover, they can train patients to adopt this strategy to manage their anxiety.
